# Consistent two-dimensional visualization of protein-ligand complex series

**DOI:** 10.1186/1758-2946-3-21

**Published:** 2011-06-24

**Authors:** Katrin Stierand, Matthias Rarey

**Affiliations:** 1 Center for Bioinformatics (ZBH), University of Hamburg, Bundesstraße 43, 20146 Hamburg, Germany

## Abstract

**Background:**

The comparative two-dimensional graphical representation of protein-ligand complex series featuring different ligands bound to the same active site offers a quick insight in their binding mode differences. In comparison to arbitrary orientations of the residue molecules in the individual complex depictions a consistent placement improves the legibility and comparability within the series. The automatic generation of such consistent layouts offers the possibility to apply it to large data sets originating from computer-aided drug design methods.

**Results:**

We developed a new approach, which automatically generates a consistent layout of interacting residues for a given series of complexes. Based on the structural three-dimensional input information, a global two-dimensional layout for all residues of the complex ensemble is computed. The algorithm incorporates the three-dimensional adjacencies of the active site residues in order to find an universally valid circular arrangement of the residues around the ligand. Subsequent to a two-dimensional ligand superimposition step, a global placement for each residue is derived from the set of already placed ligands. The method generates high-quality layouts, showing mostly overlap-free solutions with molecules which are displayed as structure diagrams providing interaction information in atomic detail. Application examples document an improved legibility compared to series of diagrams whose layouts are calculated independently from each other.

**Conclusions:**

The presented method extends the field of complex series visualizations. A series of molecules binding to the same protein active site is drawn in a graphically consistent way. Compared to existing approaches these drawings substantially simplify the visual analysis of large compound series.

## Background

Many methods in structure-based drug design, like virtual screening, scaffold hopping, and docking, are dealing with series of protein-ligand complexes. They are all characterized by several poses or ligands bound to one active site, which is, except for potential conformational flexibility, non-varying for the whole set. The comparative visual inspection of the different binding patterns is facilitated by a depiction mode, which takes the constant part into account. While in the context of three-dimensional (3D) visualization the superimposition of ligands in one graphical active site representation is common practice [1], the orientation of two-dimensional representations is often affected by the attempt to provide a planar and aesthetically ideal arrangement of all diagram elements. This leads to a heterogeneous overall picture within a complex series and makes the comparison of the binding modes difficult.

An approach for the 2D depiction of protein-ligand complex series with an automatically generated consistent layout of the residues for all diagrams was introduced in the software MOE [2] in 2007. The built-in 2D drawer is able to deal with single proteins, which contain multiple ligands, as well as with multiple members of one protein family. Generally speaking, the layout generation is done in two steps: First, the planar ligand diagrams are aligned in their original 3D position and this alignment is transformed to the x-y-plane. In a second step, the residues are placed based on pseudo-atom positions on a grid, which are derived from the superimposed ligands. Although the method works well in practice, the protein amino acids are represented as spherical objects only such that the individual hydrogen bonding pattern cannot be derived from the figure.

Also Ligplot [3] can be used to depict series of complexes with a consistent 2D layout for all drawn residues. In this case, the layout generation is a semi-automatic process: while the initial diagram layouts are generated automatically, the user has to choose one of them as template and subsequently to align the residue centroids manually by means of certain meta-files.

In this work, we will present an extension of PoseView [4-6] that generates series of complex diagrams with a consistent receptor layout. In its previous versions, PoseView generated automatically 2D layouts for single protein-ligand complexes by means of a ligand centered algorithm. The objective of the new approach is to find a global position for each residue providing an intersection-free arrangement of directed interactions for each individual complex diagram. In contrast to the algorithm for single complexes the new methods take the 3D arrangement of the residues into account assuming that some of the 3D adjacencies can be conserved. Therefore, these adjacencies are used as basis for the initial 2D residue arrangement around the ligand.

## Methods

**Figure 1:**
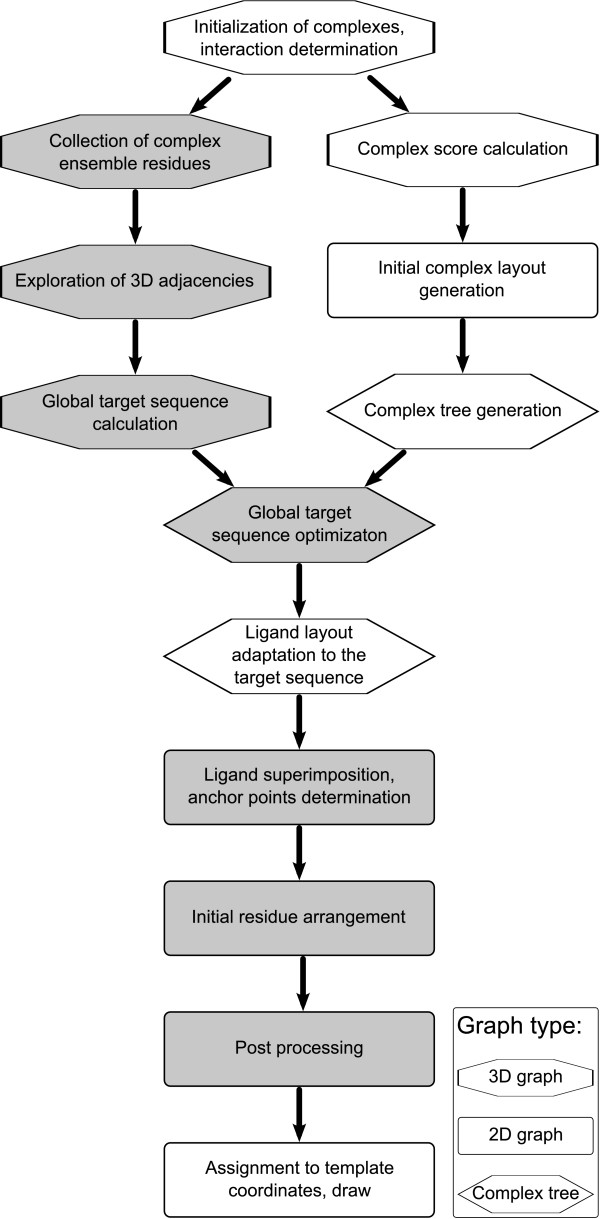
**PoseView work flow**. The PoseView algorithm proceeds on different graphs which is represented by the different shapes of the text boxes. Calculation steps based on the 3D complex representation are denoted by an octagon, the ones based on the topological tree representation are denoted by a hexagon and structure diagram based algorithms are denoted by rectangles. Additionally the steps are subdivided in those which address the global structure (gray background) and those which modify the individual complexes (white background).

In this section, we will give an overview of the whole layout generation procedure and explain the algorithms, which differ from the layout generation algorithm for single complexes. A more detailed description of the underlying PoseView methodology can be found in [4-6]. Below, these methods are summarized, where necessary for the understanding of our new approach. Additionally, a graphical representation of the work flow is given in Figure 1.

The algorithm starts with the determination of the interactions between the different ligands and the receptor and the initialization of the individual molecules. For each individual complex, a drawing complexity score and an initial layout is calculated based on the structure diagrams of the ligand and residue molecules. Furthermore, a tree is derived from the complex' connectivity. Subsequently, the residues, which are part of any of the complexes in the series, are collected and stored in the global template. Then, a global layout is computed for the diagrams of the selected residues starting with the determination of the optimal global target sequence (circular order of amino acids around the ligand) derived from the original 3D residue adjacencies. The global target sequence is optimized by means of the individual trees which are derived from the complexes. Subsequently, the individual ligand layouts are modified and their interaction atom arrangements are adapted to the global target sequence. The global layout is computed based on the convex hull [7] of all superimposed ligand diagrams and the resulting global interaction starting coordinates, called anchor points. This layout generation includes an initial placement of each residue diagram and a subsequent post optimization analog to the residue layout calculation for single complexes. In a last step, the global amino acid atom coordinates are assigned to the individual complexes and then they are drawn.

The algorithm employs three different graphs as underlying data structures:

• the local 3D graphs which are composed of the ligand and the interacting residues for each individual molecular ensemble

• the corresponding 2D graphs containing the coordinate sets of the molecular structure diagrams

• a tree for each complex, containing only topological information based on the connectivity of the individual molecular ensembles

In Figure 1 the methods are labeled according to the type of graph underlying the calculation step.

### Terms and definitions from the layout generation for single complexes

Before starting the algorithm description, some of the basic terms, which are defined in previous papers and used in the following section, will be mentioned here:

• The order of ligand interaction atoms that is generated by a circular walk around the ligand is referred to as *interaction atom order*.

• A *good layout *is characterized as an arrangement of all depicted complex elements that, on the one hand, is collision free and on the other hand fulfills aesthetic and chemical structure diagram conventions. The quality of the complex diagram layout results from the combination of an intersection-free interaction atom order, the convenient geometric positioning of the single structure diagrams (SD), and the consequential arrangement of interaction lines.

• Each residue in a complex has a *main interaction direction *with a defined starting point. It is the resultant from the individual optimal directions of all directed interactions which are connecting one residue with the ligand. For each molecule of the complex ensemble, the individual directions are derived from the convex hull of the 2D atom coordinates which leads to a radial orientation and avoids collisions of the structure diagram and its interaction lines. The main direction is calculated for both the ligand and the residue. The centroid of the corresponding interacting atoms is defined as the *main interaction direction starting point*. The placement of a residue is realized by superimposition of the matching ligand and residue main interaction vectors.

### Initialization of complexes and interaction determination

The input and initialization of the individual complexes is performed using the chemistry model and file handling utilities implemented in FlexX [8]. The interactions can originate from either the built-in geometry-based interaction model in PoseView [4] or calculated by any other software. In the case of external interaction calculation, the interactions have to be defined in the comment block of an input file in mol2 format. If the interactions are determined by the PoseView model and the protein is defined in a PDB file, the separation of residues from the 3D structure is done as described previously [6].

### Complex score calculation

Before starting the layout calculation, the complexes are scored and ordered according to their drawing complexity, which is determined mainly by the number of interacting residues and the number of residues with more than one directed interaction to the ligand. The latter ones are responsible for the need to potentially modify the initial ligand structure diagram layout in order to provide an intersection-free arrangement of interaction lines. Therefore, the complexes are ordered according to the following score *σ*:

where #*interactions_i _*is the number of directed interactions from residue *i *to the ligand.

In case of greedy layout decisions in the context of the sequential calculation steps like the determination of ligand anchor point coordinates, the scoring ensures that the more complicated complexes are treated first. The subsequent ligand superimposition method as well as the calculation of the initial global residue sequence take advantage of this ordering.

### Initial complex layout generation

For each complex of the series, the structure diagrams of all interacting ligands and residues are initially generated as basis for the following steps. At this point, no optimization procedures are performed even though collisions may occur and the interaction atom order may be suboptimal. The resulting 2D coordinates are used as starting point for the following algorithms.

### Representation of single complexes as a tree

A rooted tree, in the following referred to as complex tree, is derived from the initial complex layout whose nodes represent atoms or groups of atoms like non-interacting ring systems and whose edges represent a covalent bond or interaction. This leads to a uniform representation of all complex parts - ligand atoms and bonds, interactions, residue atoms and bonds - and permits on the one hand a condensation of parts of the complex, which are irrelevant for layout decisions, and on the other hand an ordered layout processing. Both features improve the average run time in comparison to the full enumeration of possible bond modifications on the basis of the structure diagram representation. The tree is directly derived from each individual complex, reflecting the relative 2D arrangement of structure diagram elements by its edge sorting. Subsequently, it is processed in order to simplify the following layout generation process under conservation of all chemical and topological information that is needed to generate valid 2D layouts. Initially, for each atom a node is inserted and these nodes are connected by edges according to the connectivity in the original protein-ligand complex by the covalent bonds and interactions. Unlike acyclic parts of the structure diagram, rings are represented by a single central node such that circles are avoided. Additionally, for each ring atom that is starting point of a substituent or an interaction, an additional node is inserted and an edge, that connects this new node with the center node.

The residue part of the complex is represented only partly in the tree: In contrast to the ligand, whose atoms are all considered in the tree generation, the residue atoms are only included if they interact with any ligand atom. Hence, all residue atom nodes are leaves of the tree. For directed interactions between ligand and residue three different layout scenarios are possible: In the first case, there is only one interaction between both molecules such that one node has to be inserted in the tree to represent the residue part. In the second case, one atom of the residue forms more than one interaction to the ligand. This leads to a representation of this one atom by multiple nodes in order to avoid circles in the graph. Such nodes get an adjacency label, that is realized by the global interaction order sequence as described in the following subsections. During the subsequent interaction order optimization, the method tries to find an order, which satisfies this adjacency demand. The third case offers two distinct atoms of the same residue interacting with the ligand. This is also solved by inserting multiple nodes and setting an adjacency label. Hybrid forms are treated the same way.

**Figure 2:**
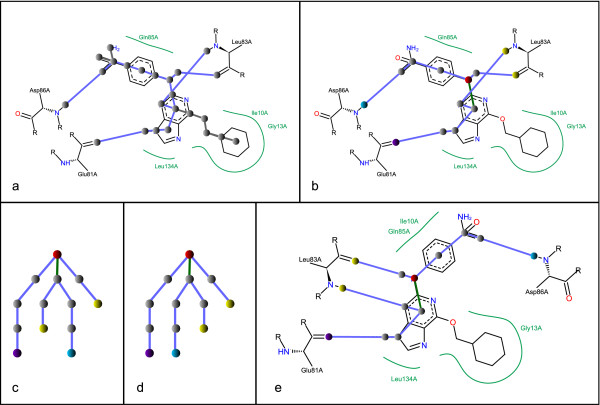
**Complex tree generation**. a) In the initial complex tree, a node is inserted for each acyclic atom, for each ring center, for each ring atom that is either an interacting atom or a substituent starting atom, and for each amino acid interaction atom. Subtrees which lie not on a path between interaction atoms are denoted by gray edges. b) After edge condensation and removing subtrees which are not needed the resulting tree contains only one rotatable edge which is highlighted in green. The root node is colored red and the leaves are colored accordingly to the amino acid they belong to. c) The derived complex tree has a suboptimal leaf order, which is optimized by rotation d) such that the yellow nodes are adjacent. e) The tree modifications are applied to the ligand structure diagram.

The edges are labeled with modification operations depending on the related bond or interaction (for the assignment procedure of bond modifications see [6]). Due to the nature of 2D bond rotations, which are the equivalent of 180° flips, their effect on the interaction atom order is canceled by a second rotation of any other edge that influences the order of the same interaction atom range. Therefore, subsequent edges of the tree, whose rotations cause the same layout modification, can be condensed to single edges. The number of potential modifications for ligands with long carbon chains for example can be significantly decreased: if five adjacent edges are merged, the number is decreased from 2^5 ^to 2 potential modifications. Branches, which represent non-interacting molecule parts and cannot influence the interaction atom order, are cut. Based on this condensed tree, a root is calculated by determination of the central node: Starting at each leaf node, a depth first search is performed and the nodes are labeled according to their depth in order to find the longest path in the tree. The node that lies in the middle of the longest path is selected as root node. Now, edges can be directed in root-to-leaf order and sorted according to the polar angle between the corresponding bonds and the bond representing the unique edge to the parent node. This results in a tree, whose leaf order is equal to the order of residues around the ligand in the complex. The number of leaves is equal to the number of directed interactions. A graphical example for the tree generation and layout modification is given in Figure 2.

### Collection of complex ensemble residues

Beyond the optimal individual layout, a good global layout that compromises with all individual optimal layouts has to be computed. The generation of such a layout starts with the collection of all different interacting residues based on the individual complexes. For all single complexes the interacting residues are enumerated and, if not already found in a previous complex, a 3D representation is added to the global template structure. The mapping of equal residues is realized by comparing their 3-letter code, their sequence number and chain ID. Subsequent to the collection, the structure diagrams of all residues are generated and also stored in the template.

### Exploration of 3D adjacencies and global target sequence calculation

For many complexes more than one ligand structure diagram layout provides an intersection-free arrangement of diagram elements. As parts of the ligands in their bound conformation are planar or rigid because they consist of ring systems or non-rotatable bonds, 3D residue adjacencies are a good heuristic starting point to calculate the global initial interaction order, also called global target sequence. In this step, distances and adjacencies of the 3D residues are computed along the active site molecular surface [9] in order to find an adequate circular arrangement of the residues around the ligand. Walking along the surface is necessary because at narrow points in the binding pocket the direct distance between two residues on either side of the lumen is relatively small in comparison to the path length along the surface. In this case, using the surface path as distance function is more suitable, because it takes account to the fact that the ligand lies between both residues and that they are therefore not adjacent. A surface triangulation [10] is used as basis for the path calculation and a breadth first search [11] is performed starting at each residue that is member of the complex ensemble. A Hamiltonian cycle of all complex ensemble residues is calculated for the resulting complete adjacency graph by using an approximation to the minimal spanning tree [12]. The order of residues in the Hamiltonian cycle is used as initial global target sequence.

### Global target sequence optimization

A global target sequence represents the order of all interacting residues available in the template structure whereas a local target sequence is derived from the global target sequence by deleting all residues which doesn't take part in the formation of the currently processed complex. An optimal global target sequence is characterized by an intersection-free matching of all individual residue sequences of the different complexes. The initial global target sequence is therefore subsequently optimized under consideration of the first *n *ligands of the complex series by checking if their interaction atom order can be modified via edge modifications such that an intersection-free matching to the global target sequence is possible. The default value of *n *is set to 20. In case all complexes can be drawn with an intersection-free matching, the algorithm stops. Otherwise, the sequence of residues is changed randomly and tested again. The acceptance of a new order is controlled by a Simulated Annealing method in order to avoid getting trapped in local optima in terms of increasing numbers of intersections.

### Ligand layout adaptation to the target sequence

**Figure 3:**
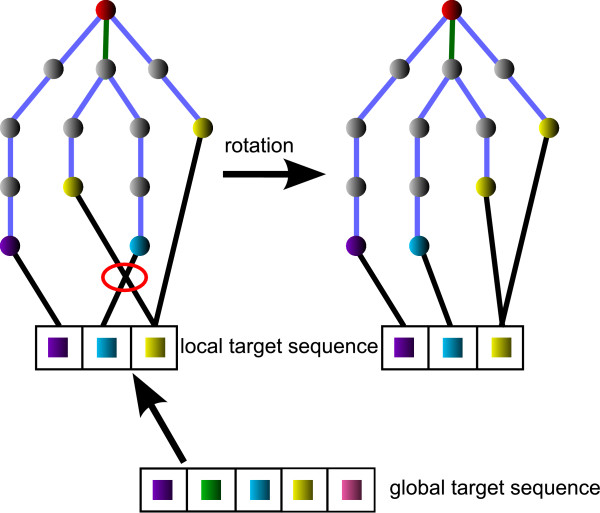
**Target sequence alignment**. The global target sequence contains one entry for each residue being part of the series complex ensemble. A local target sequence is derived by deleting all entries which have no corresponding residue in the individual complex in question. After inserting the matching edges (black), they are searched for intersections. By modification of the complex tree edges (blue and green), in this case a rotation of the green edge, the intersections are removed.

**Figure 4:**
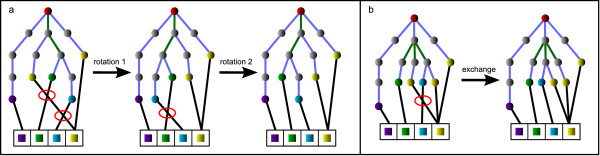
**Tree modifications**. A rotation of an edge (a) inverts the leaf order of the whole subtree. A rotation of the rotatable edges in the relevant subtree compensates this inversion. In case of exchanges (b) the leaf order in the subtrees is not changed.

As previously described, the residue sequences in the single complexes are represented by the leaf order of the complex trees and a tree can feature more than one leaf per residue. The leaf sequences are iteratively matched to the local target sequence, which are derived from the optimized global target sequence, in the order that was calculated during the complex scoring. In contrast to the ligand-centered method, which was applied in case of single complexes [6], the ligand has now to be fitted in a given residue arrangement. Therefore, the leaf order of the tree has to be modified by rotating or exchanging edges until it matches the residue order in the local target sequence. The matching is realized by inserting additional edges, one from each leaf node to the position of the corresponding residue in the local target sequence, see Figure 3. In some cases, more than one edge leads to the same position in the target sequence owing to the occurrence of multiple leaves representing the same residue. As the fitting part, intersections in the matching are tried to be solved by modifying the tree applying the available modification operations. To solve a matching intersection, the common parent edge of the two appropriate leaf nodes is selected and, if possible, modified. In case of a rotation, all sub-trees containing rotatable edges have to be rotated back in order to keep the sequence valid and to affect only the relative order of the two matching edges in question. In contrast to this, exchanges of edges, which are descending from the same node, do not invert the leaf order and the sub-trees stay untouched, see Figure 4.

### Ligand superimposition and anchor point determination

Beginning with the superimposition of ligands all following layout generation steps are based on the precalculated 2D structure diagram information. The placement starts with the ligands by determination of the anchor points for the different residues. The term anchor point is defined as the global coordinate for the starting point of the main interaction direction of a residue on the ligand side. Thus, the number of anchor points is equal to the number of residues in the global structure. Corresponding to the anchor point, each residue features a global residue coordinate, which defines the global starting point of the interaction main direction of all interactions starting at this particular amino acid in any of the complexes. The computation of the global residue coordinate will be described in the following paragraph. The ligand anchor points are calculated by iteratively superimposing the ligands of the single complexes according to the order that is defined by the complex scoring. The first ligand is translated such that its centroid lies in the origin. Then, for each residue that is interacting with this particular ligand, the main interaction direction starting point is calculated and stored as the anchor point. All other ligands are superimposed to the firstly placed ligand by minimizing the RMSD between the common subset of own and already placed template anchor points. If in the course of the superimposition new anchor points are placed, they are assigned to the appropriate residue in the global structure.

### Initial global residue arrangement

Similar to the method for single complexes, the global positioning of the residue structure diagrams is based on a convex hull, but the underlying point set is, unlike in the case for single complexes, derived from the superimposed anchor points. The convex hull is represented as a circular path consisting of directed edges. Hence, each node has one incoming and one outgoing edge. To each anchor point, an edge of the convex hull is assigned: If the anchor point is a convex hull vertex, the edge leading to this vertex is chosen; otherwise the edge with the smallest distance to the anchor point in question is selected. From all interaction main directions of the individual complexes calculated in the initial complex layout generation, the overall main direction is chosen to be the median when sorting their directions by the polar angle to the corresponding edge of the convex hull. The global residue coordinate is set to a point on this straight line with a distance of five standard bond length from the anchor point. The adjustment of the residue structure diagram is done by superimposing the global main direction of the ligand and the inverted resultant direction of all individual residue interaction directions of the individual complexes.

### Global layout post optimization

Analog to the generation of single complexes, the initial placement may cause collisions. These are handled with an approach that is in principle the same as described before [6]. The major difference is that the collision detection is not performed on basis of atom and bond coordinates but by testing for overlaps between the convex hulls of the global residue structure diagrams and the convex hull of ligand anchor points respectively, because the atom-wise comparison would slow down the collision handling significantly. Additionally, intersections of interaction lines as well as intersections crossing convex hulls are detected.

### Drawing the complexes

Subsequent to the global layout generation, the coordinates are assigned to the single complexes of the series. The ligand is drawn superimposed to the corresponding anchor points and the amino acids are drawn at their global positions. The interaction atoms of the ligand are not necessarily identical with the anchor coordinates. Thus, the interaction lines have to be adapted to the local complex coordinates. In a final step, the hydrophobic contacts are placed and drawn; they are not part of the global layout.

## Results

The new method was applied to different test sets. In the following, three examples will be presented: two of them feature different ligands bound to the same protein (PARP and UK) while the other data (ER*α*) is composed of different crystal structures from the PDB [13] with an individual protein file for each of the complexes. Based on the presented application examples, the strength and weaknesses of the new approach will be discussed.

Before starting the layout calculation, the complexes with only one directed interaction or without directed interactions are removed from the sets as well as duplicates. Complexes are recognized as duplicate if their ligands and the interaction patterns are identical. A prerequisite for a successful layout generation process is that all ligands are bound to the same active site and protein chain; otherwise no common residues can be found by the algorithm and the layout alignment fails. In all examples the complexes are sorted according to their score, such that the ones with the highest number of interactions come first.

### Poly ADP Ribose Polymerase (PARP)

**Figure 5:**
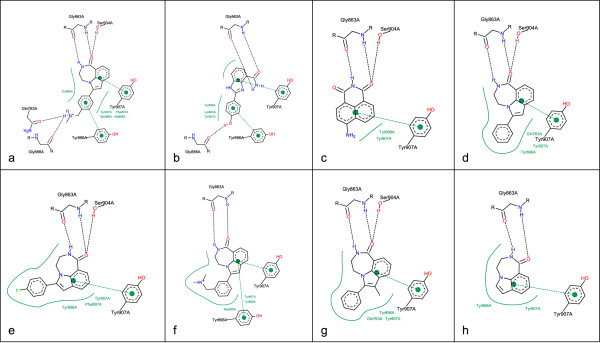
**Aligned visualization of Poly ADP Ribose Polymerase complexes**. The ligands were taken from the ZINC database [14] and docked to a protein provided by the PDB (PDB code: 1EFY).

The ligands for the PARP example (Figure 5) were taken from the ZINC database [14] and docked to a protein provided by the PDB (PDB code: 1EFY). The complex ensemble consists of six different residues; two of them (Gly 863A and Tyr 907A) are represented in each complex. Due to the similarity of the ligand shapes and the interaction pattern the resulting overall picture makes the differences between the complexes readily identifiable. The orientation of the ligand in the different drawn complexes is only dependent on the position of the residues. In series with very similar ligands, like in this case, a ligand based alignment would further improve the comparability between complexes.

While in Figure 5b the order of interaction atoms is properly aligned to the target sequence, a collision is caused by the ligand layout. This could be avoided by an additional ligand layout post optimization step that searches for alternative ligand layouts, which improves the geometric arrangement of ligand interaction atoms without affecting their topological order. In this case, flipping the upper ring system and rotating the amide group would remove the collision between interaction lines and the ligand structure diagram. For all other complexes, a collision-free layout could be generated.

**Figure 6:**
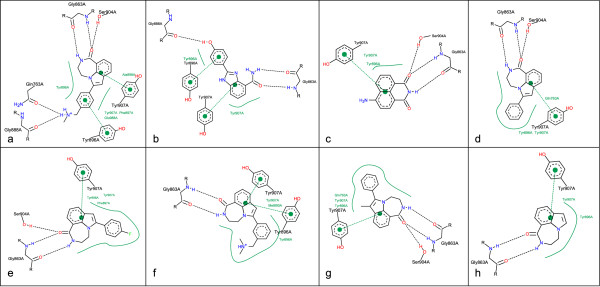
**Visualization of Poly ADP Ribose Polymerase complexes with default orientation**. The depicted complexes are identical to the protein-ligand complexes in Figure 5, but their layout is calculated independently from each other.

Figure 6 shows the same series like Figure 5, and the complexes are in the same order but in contrast to the previously shown depiction the complexes are drawn independently from each other in their default orientation. In this case, the similarity is not as obvious as in the aligned visualization with a consistent residue layout.

### Estrogen Receptor α (ERα)

**Figure 7:**
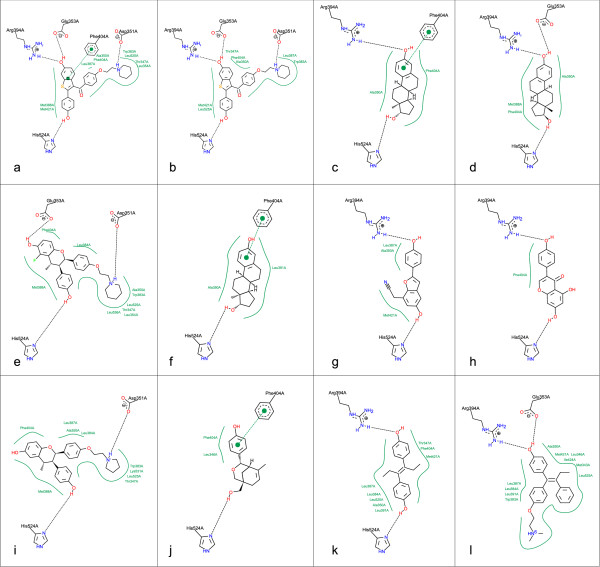
**Aligned visualization of Estrogen Receptor ***α ***complexes**. The diagrams show complexes of proteins with co-crystallized ligands with the following PDB accession codes: 1ERR (a), 2JFA (b), 1G50 (c), 1PCG (d), 1YIN (e), 1QKU (f), 1X7E (g), 1X7R (h), 1YIM (i), 2B1V (j), 3ERD (k), 3ERT (l).

In contrast to the former example, the ligands of ER*α *feature a more heterogeneous picture (Figure 7). The data set is an representative collection of crystal structures from the PDB (PDB codes, ordered according to the complex scoring of PoseView: 1ERR, 2JFA, 1G50, 1PCG, 1YIN, 1QKU, 1X7E, 1X7R, 1YIM, 2B1V, 3ERD, 3ERT). Nevertheless, an overlap-free layout with a consistent residue layout could be computed. The differences in size, orientation and shape of the ligands make the comparison between binding modes difficult. In this case, an uniform scaling as well as drawing all residues of the complex ensemble in each diagram independently from the existence of an interaction with the ligand would enhance the legibility. The atoms of the residues are uniquely named accordingly to their identifier in the PDB file. In Figure 7a, b, d, e, and 7l the carboxylate of Glu 353A is hydrogen bonded to the hydroxyl group of the ligand. Due to its conformational flexibility, the interaction switches from one to the other carboxyl oxygen between the different complexes. 7a (1ERR) and 7b (2JFA) contain both raloxifene as ligand but differ in the existence of a π stacking interaction to the Phe 404A residue. The distances of the ring centers between ligand and amino acid vary such that in the first case it is within the default distance threshold of 5Å for interacting ring centers of a t-shaped *π *stacking interaction and in the second case it is not.

### Urokinase (UK)

**Figure 8:**
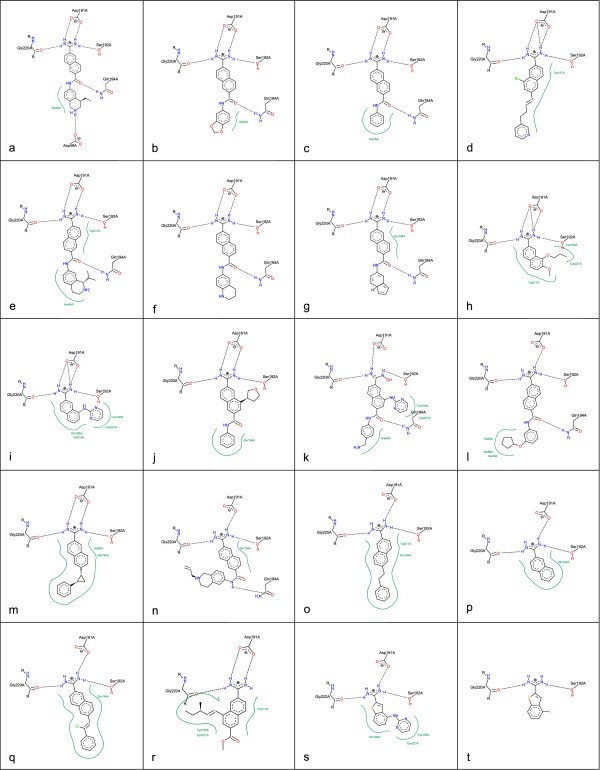
**Aligned visualization of Urokinase complexes**. The ligands are taken from a dataset provided by Brown and Muchmore [15]. The protein file is stored in the PDB with the accession code 1OWK.

The third example consists of a randomly selected subset of the UK complex series provided by Brown and Muchmore [15] (different ligands bound to 1OWK), Figure 8. Here, the binding mode shows only minimal variations and is characterized by a salt bridge between Asp 191A and an amidinium group of the ligand. The remaining hydrogens of this amidinium group form a hydrogen bond to Gly 220A on the one side and Ser 192A on the other side of the Asp191A. Figure 8o shows a disadvantage of the template based orientation of the residues: contrarily to all other complexes the Gln 194A has an acceptor function but the side chain oxygen points not towards the ligand and a collision occurs. In case of flexible side chains and different possible protonation states a compromise between a consistent layout and flexibility in order to avoid collisions is necessary.

## Discussion

We have implemented an extension of the PoseView algorithm that automatically generates consistent residue layouts for series of related complexes with different ligands bound to one protein. The layout generation is performed receptor-based taking into account the 3D residue adjacencies as well as the ligand topology. If not defined, the interactions and the resulting complex ensemble can be determined during run time.

All presented test sets feature a good overall layout quality that is comparable to the results of the PoseView version for single complexes. The ligand and the residues forming directed interactions are drawn in atomic detail as structure diagrams and arranged such that the visualized complexes are mainly collision free. As intended, the comparability and legibility within a complex series was considerably improved due to the consistent residue layout. While the residue orientation is fixed the ligand orientation changes over the different diagrams. An example can be found in Figure 7b and 7i. This is caused by the difference in the interaction patterns and the minimization of the deviation between the optimal interaction directions of the ligand and the real interaction directions given by the globally set amino acid positions. In contrast to known methods [2,3], this approach combines a high degree of detail considering the IUPAC structure diagram conventions with the independence from any particular interaction model. An unsolved challenge is the handling of different protonation states and side chain orientations within one series. Also the depiction of residues which form no interactions to the particular ligand, for example colored light grey, would enhance the readability.

In summary, the presented method extends the field of complex visualization. The aligned depiction of related complexes in atomic detail offers the possibility to get a quick insight in the differences and similarities within a series.

## Competing interests

The authors declare that they have no competing interests.

## Authors' contributions

KS developed, implemented and tested the presented method. KS prepared the manuscript for this publication. MR supervised and coordinated the project. Both authors have read and approved of the final manuscript.
